# Negative effects of restricted sleep on facial appearance and social appeal

**DOI:** 10.1098/rsos.160918

**Published:** 2017-05-17

**Authors:** Tina Sundelin, Mats Lekander, Kimmo Sorjonen, John Axelsson

**Affiliations:** 1 Department of Clinical Neuroscience, Karolinska Institutet, Stockholm 17177, Sweden; 2 Department of Psychology, Stockholm University, Stockholm 10691, Sweden; 3 Stress Research Institute, Stockholm University, Stockholm 10691, Sweden

**Keywords:** sleep, sleep restriction, faces, attractiveness, social appeal, sleepiness

## Abstract

The importance of assessing evolutionarily relevant social cues suggests that humans should be sensitive to others' sleep history, as this may indicate something about their health as well as their capacity for social interaction. Recent findings show that acute sleep deprivation and looking tired are related to decreased attractiveness and health, as perceived by others. This suggests that one might also avoid contact with sleep-deprived, or sleepy-looking, individuals, as a strategy to reduce health risk and poor interactions. In this study, 25 participants (14 females, age range 18–47 years) were photographed after 2 days of sleep restriction and after normal sleep, in a balanced design. The photographs were rated by 122 raters (65 females, age range 18–65 years) on how much they would like to socialize with the participants. They also rated participants' attractiveness, health, sleepiness and trustworthiness. The results show that raters were less inclined to socialize with individuals who had gotten insufficient sleep. Furthermore, when sleep-restricted, participants were perceived as less attractive, less healthy and more sleepy. There was no difference in perceived trustworthiness. These findings suggest that naturalistic sleep loss can be detected in a face and that people are less inclined to interact with a sleep-deprived individual.

## 1. Introduction

Telling someone they look tired says more about your perception of them than you might think. Certain malleable facial and bodily cues affect how people are perceived, such as skin tone and walking speed for health [1,2] and eyelid openness for attractiveness and intelligence [3]. A sleep-deprived, tired-looking face, with dark circles under the eyes and swollen eyelids [4], is perceived as less attractive and less healthy [5]. Since humans have a tendency to ascribe positive qualities to people considered attractive, especially interpersonal qualities such as sociability and interpersonal competence [6,7], you may even be suggesting that you are less interested in being around the tired-looking person. Furthermore, in this case, you might be making the right decision; sleep-deprived individuals report being less optimistic and sociable [8], they are worse at understanding and expressing emotions [9–11], less empathetic [12,13] and more prone to accidents [14]. A non-alert face may thus act as a cue to others about suboptimal functioning in social situations. The ability to perceive and process this information from such superficial cues would therefore serve an adaptive function.

But attractiveness is not the only factor that could influence others' behaviour towards someone with sleep loss. Fatigue is a canonical symptom of poor health [15], and sleep disturbances are typically comorbid with both somatic and mental ill-health [16]. Sleep-deprived individuals also look less healthy [5], and humans, like many other animals, tend to be disease avoidant. If someone appears to be contagious, others are likely to keep their distance [17]. Specifically, it has been found that, when exposed to disease primes, through pictures and information about contagious disease, people rate themselves as less extroverted and are faster at avoidant responses to facial photographs [18]. Having an unhealthy-looking face, whether due to sleep deprivation or otherwise, might thus activate disease-avoidance mechanisms in others and render one's surroundings less socially inclined. Although attractiveness may only be weakly related to actual health (see e.g. [19] for review), faces that look healthy are also considered attractive [5,20,21]. Since sleep-deprived faces are both less attractive and less healthy-looking than their well-rested counterparts, they contain at least two perceptible features possibly impacting others' willingness to socialize with them.

Evaluation of people's faces generally occurs along two dimensions, valence (negative–positive) and dominance (submissive–dominant), where trustworthiness and attractiveness are highly correlated with the valence dimension [22]. Considering the relationship between trustworthiness and attractiveness, it is possible that attractiveness is not the only factor on the valence dimension that is affected by sleep loss; a sleep-deprived face may be regarded as less trustworthy as well. It has also been suggested that valence can be seen as a signal of whether to approach or avoid a person [22], meaning that if a less trustworthy appearance follows sleep loss, this would further decrease others' approach intentions toward someone who has not slept.

Previous research has shown that one night of partial sleep restriction followed by 31 h of wakefulness resulted in a less attractive, less healthy and more tired appearance [5]. However, in everyday life, it is more common to experience partial sleep loss than total deprivation of sleep. The question thus still remains whether appearances are affected by more natural sleep loss. One indication is given by the change in appearance of patients photographed before and after being treated for obstructive sleep apnoea. After at least two months of treatment, they looked more alert, more youthful and more attractive than when their sleep was still notably disturbed [23]. Being a clinical group though, it is hard to ascertain whether it was just better sleep that caused the difference or whether other health factors came into play. It has also been shown that women with chronic poor sleep rate themselves as less attractive than women with normal sleep [24], but again, it is unclear whether reduced sleep is actually the main cause, and whether this self-evaluated attractiveness is related to how others perceive them.

### 1.1. Purpose of the study

The purpose of this study was to investigate whether people are less willing to socialize with someone who has not slept, and whether a more naturalistic sleep restriction—4 h in bed for two nights—is enough to affect how one is perceived, specifically regarding the attractiveness, perceived health and sleepiness of someone's face. Taking this exploration one step further, we wanted to know whether a decreased willingness to socialize with sleepy people might be based on their being less attractive and looking less healthy, or possibly less trustworthy. A secondary purpose was to replicate the findings that a tired appearance is related to looking less healthy and less attractive [5]. This time, however, the focus was on the more precise concept of ‘sleepiness’ rather than the more general ‘tired’ or ‘fatigued’. Since tiredness can be indicative of physical and/or mental fatigue rather than sleep propensity [25], the aim was to investigate whether sleep loss also affects others' perceptions of someone's sleepiness, and whether this is related to attractiveness and health.

## 2. Material and methods

### 2.1. Participants

Two sets of participants took part in this study, those who were photographed (subjects) and those who rated the photos (raters).

#### 2.1.1. Subjects

The photographed subjects were 25 healthy participants (14 female; mean age = 23.9, s.d. = 5.9) recruited at major universities in the Stockholm area. Thirty-eight out of 63 potential participants were excluded due to having a sleep need other than 7–9 h per night, having insomnia or other sleep problems, having health problems or not being available on study days. Other exclusion criteria were smoking, shift work during the previous three weeks and excessive coffee intake (more than five cups per day and problems abstaining). Subjects received financial compensation for their time.

#### 2.1.2. Raters

The raters were 122 individuals (65 female, 56 male and one who did not disclose gender; mean age = 30.8, s.d. = 13.3), naive to the purpose of the study, recruited from the general public in Stockholm. In order to participate, raters had to be between the ages of 18 and 65 and not a student of psychology. They received a movie ticket for their participation.

### 2.2. Materials and procedure

#### 2.2.1. Photographs

Subjects came into the laboratory on two occasions: once after being instructed to sleep for 8 h for two consecutive nights and once after only being allowed 4 h in bed for two consecutive nights. These visits were in a counterbalanced order, at least one week apart. To verify adherence to the sleep protocol, subjects used actigraphs (Camntech, AW4, Cambridge, UK), clock-sized units which measure activity and give good estimates of sleep timing [26]. Subjects were instructed to go to bed between 22.00 and 00.00 or 00.00 and 02.00, and wake up between 06.00 and 08.00 or 04.00 and 06.00, respectively, for normal sleep and sleep restriction days. The mean total sleep time per night during the normal sleep condition was 7 h 35 min, s.d. = 71 min, ranging between 6 h 43 min and 9 h 39 min. During the sleep restriction condition, the mean total sleep time per night was 4 h 15 min, s.d. 40 min, range 3 h 42 min–6 h 52 min. The average difference in hours slept per night between the two conditions was 3 h 30 min, s.d. = 47 min, indicating that subjects on average received approximately 7 h less sleep during the sleep restriction condition.

Subjects were photographed at the same time of day on both occasions (14.30 ± 1 h), with a Nikon D90 (Nikon, Tokyo, Japan) in a standardized flash setting (resolution: 4288 × 2848 pixels, white balance: 5880 K) by a photographer blind to the condition of the subject. Subjects wore the same dark grey T-shirt at both times, had their hair pulled back, and wore no make-up and minimal jewellery. They were told to sit comfortably, look straight into the camera and relax their face. A minimum of six photos were taken during each condition. The most representative photo, i.e. the one most similar to the rest of that set of photos, from each occasion was later chosen by a person blind to the conditions and the purpose of the study. Directly following the photo session, subjects rated their sleepiness on the Karolinska Sleepiness Scale (KSS), ranging from 1 (*extremely alert*) to 9 (*very sleepy*) [27]. Subjects were not informed about the purpose of the study or the photos prior to being photographed.

#### 2.2.2. Ratings

Fifty facial photos (two of each subject, one from each condition) were displayed one by one on a 17-inch computer screen in a pseudo-randomized order. The same photo could be displayed more than once, but the same subject was never shown twice in a row. The photos were 140 × 160 mm and centred, so that the pupils of all subjects were levelled. The faces were rated on seven-point scales pertaining to sociability (*How much would you like to socialize with this person? Not at all –Very much*), trustworthiness (*How trustworthy is this person? Very untrustworthy–Very trustworthy*), attractiveness (*How attractive is this person? Very unattractive–Very attractive*), health (*How is this person's health? Very poor–Very good*) and sleepiness (*How sleepy is this person? Very sleepy–Extremely alert*). The questions were presented in blocks, so that all faces were rated on one question at a time in the above order. Raters had 6 s to make a response before the next face would appear. The protocol was programmed in E-prime (Psychology Software Tools, Sharpsburg, PA, USA).

### 2.3. Analyses

In order to take into account random variations in levels between both subjects and raters, the ratings were analysed using multi-level mixed effects linear regression with two crossed independent random effects. The fixed effects represent the typical subject in the 8 h per night condition (intercept), with the slope (*b*) being the effect of sleep deprivation. The random effects concern the extent to which the subjects and raters differ from the average subject and rater. The *p*-level was set to 0.05. The strength and significance of mediated effects were calculated through 1000 bootstrapped samples, using the boot [28] package in R. 3.2.2 [29].

Data from seven raters were lost because of equipment malfunction. The sleepiness scale initially ranged from *1—extremely alert* to *7—very sleepy*, similar to the commonly used KSS [27]. However, based on feedback from several of the first 40 raters indicating confusion about their responses—that the ‘positive’ end was at 1 rather than 7—the endpoints were reversed for subsequent ratings. Therefore, the responses of the first 40 raters (all raters prior to reversal of the scale) have been excluded regarding sleepiness, leaving 75 raters in the analyses of this particular factor. Ratings on other scales were retained. Raters were also excluded on the basis of low variability in ratings, i.e. a standard deviation of less than 0.5. Such a limited use of the scale may indicate low motivation to adhere to the instructions of the task. This led to nine raters being removed for sociability, 19 for trustworthiness, seven for attractiveness, nine for health and two for sleepiness.

Since the variable ‘sleepiness’ was of interest, rather than ‘alertness’, the sleepiness scale was reversed in the analyses and figures to improve the ease of interpretation; a higher value on the scale thus means more sleepy.

## 3. Results

### 3.1. Sleep restriction

Raters were less willing to socialize with a subject who was sleep restricted (table 1). Sleep-restricted subjects were also rated as less attractive, less healthy and more sleepy compared with their well-rested selves (table 1). There was no significant difference in ratings of trustworthiness between the two conditions (table 1).

**Table 1.** Effects of two nights of sleep restriction on rated appearance. The average rating of the average face after normal sleep (*intercept*) and the fixed effect (*b*) of sleep restriction on these ratings. s.e. is the standard error. Scales ranged from 1 to 7 for willingness to socialize (*Not at all*–*Very much*), attractiveness (*Very unattractive*–*Very attractive*), health (*Very unhealthy*–*Very healthy*), sleepiness (*Very sleepy*–*Very alert*, reverse scored) and trustworthiness (*Very untrustworthy*–*Very trustworthy*).

rated factor	normal sleep (s.e.)	effect of sleep restriction (s.e.)	*p*-value
willingness to socialize	3.79 (0.13)	−0.15 (0.03)	<0.001
attractiveness	3.45 (0.16)	−0.09 (0.03)	0.003
health	4.48 (0.16)	−0.11 (0.03)	0.001
sleepiness	3.50 (0.18)	+0.25 (0.04)	<0.001
trustworthiness	4.20 (0.12)	−0.04 (0.03)	0.206

### 3.2. Other-rated sleepiness

**Figure 1.**
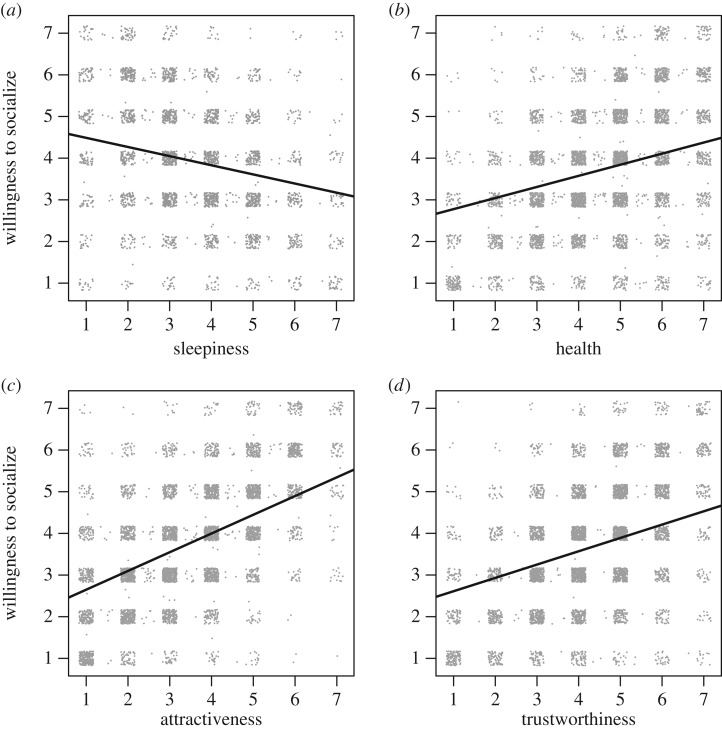
Relationship between willingness to socialize and perceived sleepiness (*a*), health (*b*), attractiveness (*c*) and trustworthiness (*d*). The dots are based on raw data, with each dot representing one rating. Jitter was applied to better illustrate the distribution of the ratings. Regression lines are from the linear mixed models, with random effects of both subject and rater.

The raters were less inclined to socialize with people who looked sleepy (*b* = −0.21, s.e. = 0.02, *p* < 0.001) (figure 1*a*). Each increment on the sleepiness scale thus corresponds to a 0.21 reduction on the sociability scale. Willingness to socialize increased when subjects looked healthier (*b* = 0.27, s.e. = 0.01, *p* < 0.001), more attractive (*b* = 0.45, s.e. = 0.01, *p* < 0.001) and more trustworthy (*b* = 0.32, s.e. = 0.01, *p* < 0.001) (figure 1*b–d*).

**Figure 2.**
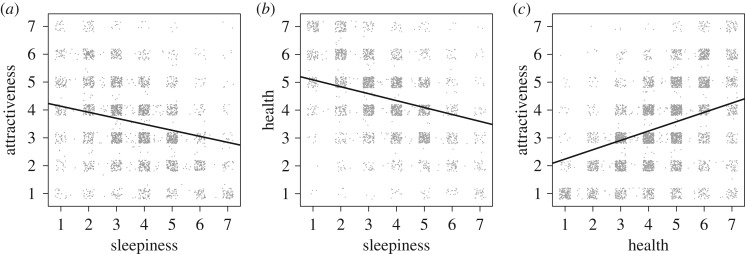
Relationship between attractiveness and perceived sleepiness (*a*), health and sleepiness (*b*) and attractiveness and health (*c*). The dots are based on raw data, with each dot representing one rating. Jitter was applied to better illustrate the distribution of the ratings. Regression lines are from the linear mixed models, with random effects of both subject and rater.

The replication analyses of the relationships between looking sleepy, attractive and healthy [5] were all significant. A sleepy appearance was related to looking less attractive (*b* = −0.22, s.e. = 0.02, *p* < 0.001), as well as to looking less healthy (*b* = −0.25, s.e. = 0.02, *p* < 0.001). A healthier appearance was also related to looking more attractive (*b* = 0.33, s.e. = 0.01, *p* < 0.001) (figure 2).

### 3.3. Mediation

**Figure 3.**
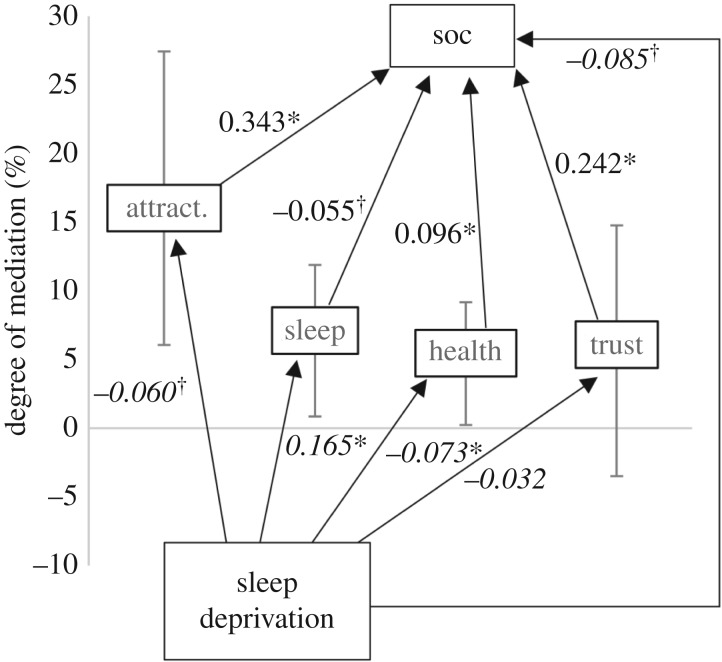
The effect of sleep deprivation on how much others wanted to socialize with the photographed person (‘soc’) and how attractive (‘attract’), sleepy (‘sleep’), healthy (‘health’) and trustworthy (‘trust’) they appeared (Cohen's *D*, in italics); the effects of attractiveness, sleepiness, health and trustworthiness on sociability (β-weights); and the mediating effects of these factors (per cent mediation). The placement of the mediator along the *y*-axis corresponds to the degree of mediation, with 95% CI. *p < 0.001, ^†^*p* < 0.05.

The average level of willingness to socialize was 0.129 (s.e. = 0.035, *p* < 0.001) standard deviations lower when subjects were sleep restricted compared with the control condition. When including other-rated sleepiness as the sole mediator in the model, the mediated effect of sleep restriction on willingness to socialize via sleepiness was −0.035 (95% confidence interval (CI): −0.047; −0.022) which is 26.8% (−0.035/−0.129, difference due to rounding) of the total effect. The corresponding values for the mediators health, attractiveness and trustworthiness were −0.019 (95% CI: −0.031; −0.007, 15.0% of total effect), −0.027 (95% CI: −0.047; −0.011, 21.4% of total effect) and −0.010 (95% CI: −0.025; 0.005, 7.9% of total effect), respectively. When adjusting for the other mediators, through including all of them simultaneously, together with sleep restriction, as predictors of willingness to socialize in a multivariate model, 7.1% of the effect of sleep restriction on willingness to socialize was mediated via sleepiness, 5.5% via health, 16.0% via attractiveness and 6.1% via trustworthiness, respectively (figure 3). The total mediated effect of sleep restriction on willingness to socialize via the four mediators was −0.045 or 34.7% of the total effect.

### 3.4. Subjective sleepiness

When subjects' sleep was restricted, they felt sleepier (*M* = 6.0, s.d. = 2.0) than when they had gotten sufficient sleep (*M* = 3.1, s.d. = 1.0; *t*_24_ = 7.5, *p* < 0.001). The sleepier subjects felt (as indicated on the KSS), the less willing raters were to socialize with them (*b* = −0.02, s.e. = 0.01, *p* = 0.029). A higher KSS score was also related to looking sleepier (*b* = 0.06, s.e. = 0.01, *p* < 0.001). There were no significant effects of the photographed subjects' self-rated sleepiness on how trustworthy (*b* = 0.00, s.e. = 0.01, *p* = 0.943), attractive (*b* = −0.01, s.e. = 0.01, *p* = 0.137) or healthy (*b* = −0.02, s.e. = 0.01, *p* = 0.083) they appeared.

### 3.5. Time awake

As subjects were allowed to regulate their own bed- and wake-times, 19 out of 25 chose to rise earlier in the morning during the sleep restriction days. This resulted in an average of 62 min (s.d. = 85 min) longer wake time before the photograph was taken. As time awake is a factor in sleepiness [30,31], additional analyses were performed in order to rule out the possibility that this was the driving factor behind the results. Adding time awake to the separate models did not affect the effect of sleep restriction on willingness to socialize or attractiveness, although it slightly decreased the effects on health (new *b* = −0.08, s.e. = 0.04, *p* = 0.041) and other-rated sleepiness (new *b* = 0.17, s.e. = 0.05, *p* < 0.001).

## 4. Discussion

When subjects had been sleep restricted before being photographed, raters were less willing to socialize with them compared with when they had gotten two nights of good sleep. Interestingly, the decreased willingness to socialize with sleep-restricted subjects was not solely due to their looking less attractive, less healthy, more sleepy or less trustworthy. These four factors mediated about one-third of the effect of sleep restriction on other's willingness to socialize with the sleep-restricted person. This suggests that sleep loss affects facial cues over and above those presently measured.

For a social animal, being left out of a group can have dire effects. Social exclusion leads to negative, even ‘painful’, emotions [32], decreased prosocial behaviour [33] and increased aggression [34]. Since sleep deprivation is implied in making people more emotionally reactive [35,36], as well as more aggressively inclined [37], these affective and behavioural responses to exclusion may be amplified in a person who has not slept, even if their immediate distress response is unaffected [38]. Whether the indicated decrease in motivation to socialize with someone who is sleep deprived actually results in overt ostracism remains to be studied. On the other hand, it may be adaptive that the cues which sleep-deprived individuals give off [4] enable them to be left alone in order to recover from their current state. Someone who is driven by the motivation to sleep could thus have less interest in socializing with other people, alternatively be less attentive to their social surroundings and as such may be less negatively affected by being undesired and excluded. If this is true, it is likely that the social effects of sleep loss found here would be stronger in real life, with both parties less motivated to socialize.

Adding to previous research on appearance after sleep loss [5], partial sleep deprivation, like total sleep deprivation, made participants appear less attractive and less healthy. Also in line with findings on how sleep apnoea and chronically poor sleep affects attractiveness [23], this is the third study to connect poor sleep to a less attractive and less healthy appearance. One possible mechanism for this change in looks is a difference in skin blood coloration. A healthy, attractive face is characterized by a certain degree of redness, which in turn is indicative of increased vasodilation and vascularization [2]. Blood flow to the skin is strongly promoted by sleep and this vasodilation may be a way for the body to facilitate the distribution of endogenous defence agents [39]. With a lack of sleep, blood flow to the skin is reduced [40], and according to raters faces look more pale after not sleeping [4].

The subjects also looked more sleepy after two nights of restricted sleep, adding to the earlier findings of people looking more tired and fatigued after a night of total sleep deprivation following a night of sleep restriction [4,5]. It is possible that the participants rating the photos would only make a distinction between sleepiness and tiredness if they were concurrently evaluated, as the two are often used as synonyms in everyday language [41]. Regardless, people seem to be able to tell when someone needs more sleep, and are more inclined to leave them alone in that case. This appears to be true even though the subjects were not perceived to be very sleepy, regardless of condition.

This study showed pictures of strangers' faces to people and asked them to indicate their willingness to socialize with the person in the picture. They had no background information about personality or interests or other factors that may affect how we perceive people. The effect of sleep restriction might be different if the raters were instead friends or family members of the photographed individual. In the case of strangers, a more pertinent question may have been ‘*Would you like to work with this person?*’ or ‘*If sick, would you like to be treated by this person?*’ because many work situations, e.g. among shift-workers in healthcare, involve tired, sleep-deprived people [42,43]. A shift in focus might also affect ratings of trustworthiness, as the context affects how different dimensions are valued [44]. In this study, there were no differences in the appearance of trustworthiness after two nights of sleep restriction, but perhaps in a different context trustworthiness would be more salient and thus more harshly judged. The fact that 16% of the raters (19 out of 122) had limited variation in these ratings indicates that they may not have felt able to make judgements about others' trustworthiness in this particular setting.

The effects of sleep deprivation on others' willingness to socialize, and the appearance of sleepiness, attractiveness and health, were arguably quite small; ranging from 0.09 to 0.26 on a seven-point scale. This is not surprising, considering the multitude of things that affect one's appearance (see e.g. [45] for a review on attractiveness). But the fact that this has been repeatedly found, despite different scales and sleep-loss paradigms [5,23], and with high power due to the total number of raters, supports these as being true effects. As with all studies where photographs are evaluated, it is hard to say whether the effects would be amplified or disguised in a real social setting. For example, one might expect a sleepy person to yawn, blink more slowly [46], have a less expressive face [11] and more monotonic speech [47]. Such multi-sensory information is generally more effective than unimodal signalling, especially when stimuli are weak [48], which speaks for detection of sleep loss being facilitated when, for example, auditory, postural and facial cues can be evaluated together. Furthermore, sleep loss in everyday life is often comorbid with stress [49] and other health issues [50], which may exacerbate the negative effects on appearance. But on the other hand, the sleep-deprived person might have put on make-up, ingested a lot of coffee and made an effort to appear more alert, which could reduce such effects. The reasons for avoiding people who look sleepy may include the fact that sleepy individuals are at a higher risk for accidents [51], or more prone to be carriers of contagious pathogens [52], or aspects making them less socially rewarding to be around. Additional research is needed to find out which cues might signal these tendencies and how they relate sleep loss to social desirability. Studying people during an actual social exchange would provide an interesting view on these phenomena.

The relationships between the different factors followed the expected patterns based on previous research. Raters were much more willing to socialize with someone they considered attractive than someone unattractive, and attractive people looked substantially healthier. Interestingly, the results also showed that between someone who looked very sleepy and someone who looked extremely alert, the change in others' ratings of both health and attractiveness increased by approximately a step and a half on the seven-point scale. These large effects were also found for others' willingness to socialize with a sleepy-looking person versus when they looked alert. Keeping in mind that these are within-subject ratings, these are notable differences and, as such, fair arguments for prioritizing a good night's sleep and using other methods to reduce a sleepy appearance before interacting with people.

A limitation with this study is the extent to which the effects can be generalized to different populations and settings. This study took place at two universities, and although the raters were of different ages and backgrounds, most of the sleep-restricted subjects were healthy students in their early to mid-20s. A further restriction is that the faces in the photographs (based on appearance and name) were mainly Caucasian. The within-subject, within-rater design and analyses give strength to the findings, but one should still be cautious about generalizations to other groups, such as people of different ages and ethnicities. Future work might be able to tell whether the effects seen here are as stable as the judgements of emotions from faces in different ethnic and cultural groups [53]. It is further likely that the vulnerability of one's appearance to sleep loss differs between individuals. Studies on such individual differences should ideally include more subjects, with a larger range than this study regarding, for example, attractiveness.

## 5. Conclusion

This study indicates that restricted sleep affects facial appearance negatively and decreases others' willingness to socialize with the sleep-restricted person. It also adds to previous studies on facial appearance after sleep loss, showing that despite using a different scale, and a less substantial and more natural sleep-loss condition, the relationships between sleep, attractiveness and a healthy appearance still hold. Future studies would benefit from looking into the mechanisms of these findings as well as investigating perceptions of, and interactions with, sleep-deprived individuals in real-life settings.

## Data accessibility

Data are available at https://osf.io/n64fm.
